# Epigenetics and Pain: New Insights to an Old Problem

**DOI:** 10.7759/cureus.29353

**Published:** 2022-09-20

**Authors:** Lisa Nirvanie-Persaud, Richard M Millis

**Affiliations:** 1 Department of Pathophysiology/Department of Medical Education, American University of Antigua, Coolidge, ATG; 2 Department of Pathophysiology, American University of Antigua, Coolidge, ATG

**Keywords:** environment-gene interaction, nociception, pain theories, analgesia, epigenetics

## Abstract

Physicians and neuroscientists have long observed that factors such as thoughts, emotions, and expectations can influence the perception of pain. Pain can be described as an unpleasant sensation that causes physical discomfort and emotional distress. It alerts an individual to seek help and is the main complaint that brings individuals to physicians. Though it is associated with probable tissue damage, such damage may be subtle, sometimes involving the release of algesic chemicals, and also influenced by attitudes, beliefs, personality, and social factors. The perception of pain may vary due to a multitude of these factors influencing the ascending sensory impulse propagation to the primary somatosensory cortex. The genetics and epigenetics of pain modulators have been previously studied, but there is a lack of application in the everyday management and treatment of pain due to the paucity of valid evidence-based data. We used the PubMed database as our primary tool for researching current literature on this topic. The MeSH terms used included: gene modification, epigenetics, genes, pain, analgesia, “types of pain, and theories of pain. The results were filtered as follows: publications within the last 10 years, generalized pain studies regarding the biopsychosocial aspect of pain, pertinent genes, and epigenetic modulation of those genes; 52 publications were selected for review. By addressing the external factorial causes and the appropriate application of epigenetic principles which affect pain perception, it is hoped that this review will motivate future advancements in the management of acute and/or chronic pain.

## Introduction and background

Physicians and neuroscientists have long observed that factors such as thoughts, emotions, and expectations can influence the perception of pain. Pain can be described as an unpleasant sensation that causes physical discomfort and emotional distress. It alerts an individual to seek help and is the main complaint that brings individuals to physicians. Though it is associated with probable tissue damage, such damage may be subtle, sometimes involving the release of algesic chemicals, and also influenced by attitudes, beliefs, personality, and social factors. The perception of pain may vary due to a multitude of these factors influencing the ascending sensory impulse propagation to the primary somatosensory cortex. The genetics and epigenetics of pain modulators have been previously studied, but there is a lack of application in the everyday management and treatment of pain due to the paucity of valid evidence-based data. We used the PubMed database as our primary tool for researching current literature on this topic. The MeSH terms used included: gene modification, epigenetics, genes, pain, analgesia, “types of pain, and theories of pain. The results were filtered as follows: publications within the last 10 years, generalized pain studies regarding the biopsychosocial aspect of pain, pertinent genes, and epigenetic modulation of those genes; 35 publications were selected for review. By addressing the external factorial causes and the appropriate application of epigenetic principles which affect pain perception, it is hoped that this review will motivate future advancements in the management of acute and/or chronic pain. 

Although pain is most often associated with underlying tissue damage, an individual's perception of the damage is affected by the individual's attitudes, beliefs, personality, social factors, and non-modifying factors like sex/gender and race/ethnicity [1]. Perception of pain can also be affected by underlying conditions such as comorbidities, mental stress, and substance abuse [2]. Genetic associations and factors are also thought to affect a person’s perception of pain by orchestrating the entire mechanism, so it has garnered much attention in recent years [3]. Indeed, genetic, and epigenetic factors in pain perception should be a subject of interest to clinicians because of their major role in treating pain syndromes, but most importantly, because of the potential risks to patients when pain treatments fail. The risks associated with under- or over-treatment of pain could be mitigated by recognizing genetic/epigenetic influences on endogenous pain modulators (e.g., opioids) and/or metabolism of analgesic medications. Acute and chronic pain management can benefit from research directed toward developing a more thorough understanding of how to apply the principles of epigenetics to pain treatment. In the United States, 100 million individuals suffer from chronic pain syndromes the treatment of which requires high and concentrated doses of analgesics [1]. Applying the principles of epigenetics to pain management may significantly improve our current pain treatment regimens [3]. This review intends to inform health practitioners about the epigenetic influences on pain modulation. An additional aim is to highlight how other factors (i.e., culture, acute illness, chronic illness) affect pain perception and their importance in pain management. 

Theories of pain 

**Figure 1:**
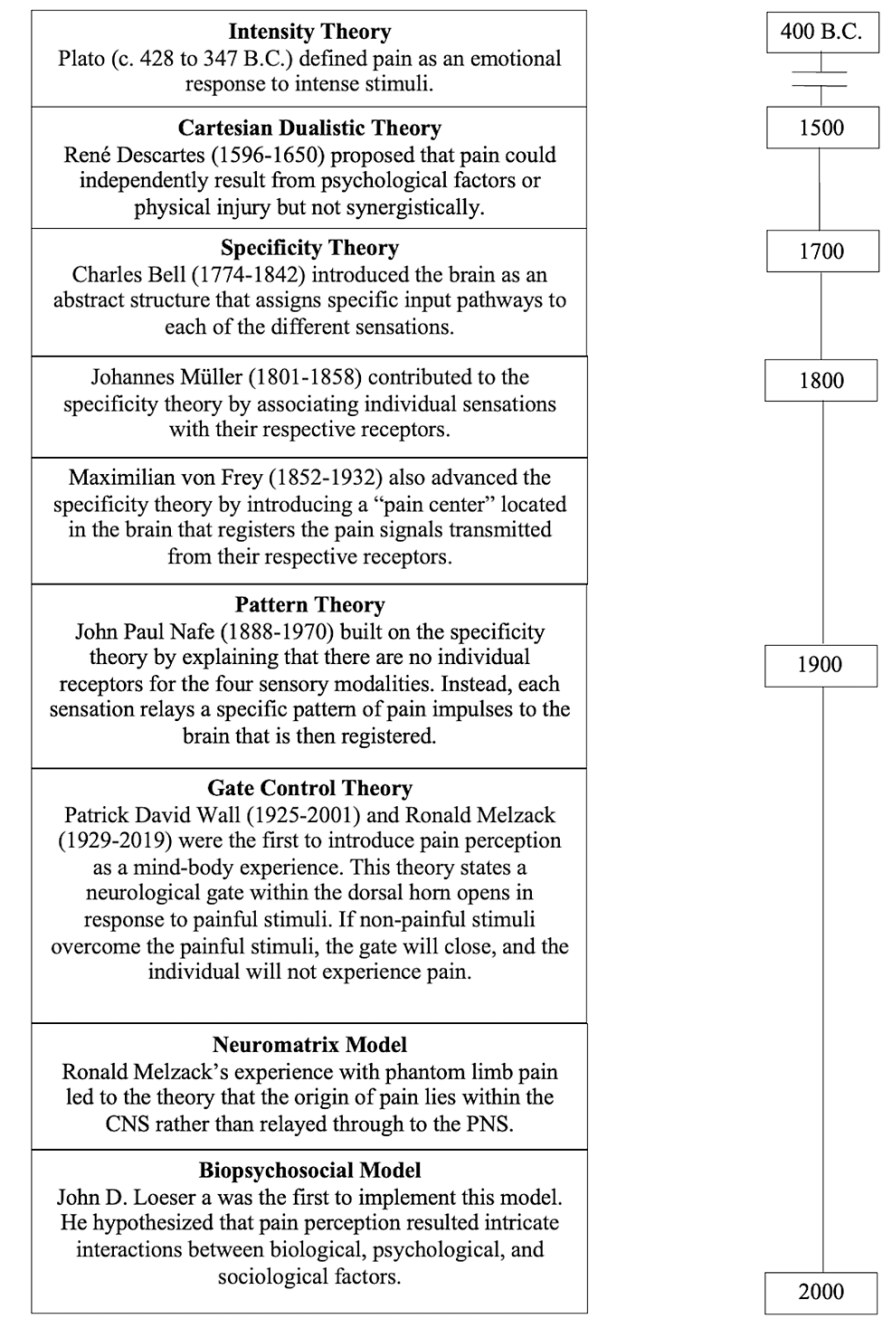
The history of pain timeline outlines the development and advancement of pain theories.

Figure 1 shows a historical timeline depicting the main theories of pain. One of the first theories proposed was by Athenian philosopher, Plato (c. 428 to 347 B.C.), who defined pain as an 'emotion' that occurs when the stimulus is intense and lasting [1]. Plato called it the “Intensity Theory.” In the early 1600s, French philosopher René Descartes (1596-1650) proposed the “Cartesian Dualistic Theory” which suggests that pain could either result from physical or psychological injury however were not influenced by each other. Thus, the belief was that physical and psychological factors were independent determinants of pain and did not combine to create a synergistic effect [1,4]. It wasn’t until the nineteenth century, after a series of experiments, that a scientific basis for pain was established. The “Specificity Theory” was initially presented by Charles Bell (1774-1842) in 1811 [1,4]. Bell’s theory allowed the brain to be introduced as a complex structure with an abstract role by presenting the idea that specific input pathways may be assigned to each of the different sensations [4]. Johannes Müller (1801-1858), a German physiologist and contributor to the specificity theory, published the Manual of Physiology in the mid-1800s [1,4]. This publication was the first to introduce the idea of individual sensations associated with their receptors [1,4]. In 1894, Maximillian von Frey (1852-1932), also a German physiologist, brought additions to advance the specificity theory which explained that pain receptors transmit signals to a "pain center" in the brain where it is then registered [1,4]. Von Frey argued that the body has a separate sensory system for perceiving pain, which contributed to the discovery of the spinothalamic tract, as well as the four separate somatosensory modalities (cold, pain, heat, touch) [1,4]. Following the specificity theory, The “Pattern Theory” was presented by American psychologist, John Paul Nafe (1888-1970), in 1929 [1]. Building on Bell’s concept, Nafe added that there are no individual receptors for the four sensory modalities, but that each sensation relays a specific pattern or sequence of impulses to the brain. The brain then correlates it with the appropriate sensation felt [1,4]. The pattern theory notion is in direct opposition to the specificity theory, and the later discovery of unique sensory receptors revealed the inaccuracy of this theory.

Gate control theory

In 1965, through experimental conduction, Patrick David Wall (1925-2001) and Ronald Melzack (1929-2019) introduced the first theory that perceived pain as a mind-body experience known as the “Gate Control Theory” [1]. This theory of pain states that a stimulus must synapse at three different locations within the spinal cord: the substantia gelatinosa in the dorsal horn, the fibers of the dorsal column, and the transmission cells in the dorsal horn [1]. The substantia gelatinosa serves to regulate the signals coming from the periphery. Here it contains the neurological "gate" that either blocks pain signals or allows them to enter the spinal cord [1,4]. If the pain is more intense than the non-painful positive stimuli, the gate will open. The signal is then modulated in the spinal cord, ascends to the thalamus where it is integrated, then registered within the primary somatosensory cortex of the parietal lobe [1]. This theory asserts that a non-painful stimulus, when greater than the painful stimuli, closes the nerve gates, preventing nerve transmission from entering the spinal cord [1,4]. A real-life example of this mechanism is exhibited within the pediatric population. In the case where a child is receiving medical care, the non-painful stimulus would be the game or play that is administered to the child at the same time as the procedure. The positive stimuli from the game or play, if high enough in intensity and profoundly distracting, close the pain gate in the spinal cord, preventing the ascending propagation of the pain nerve signal from the procedure. It is therapeutic for the child and has been implemented in healthcare facilities to aid in the delivery of medical care to the pediatric and disabled population. However, at any given time, if the pain signal overcomes the level of intensity required to open the gate, the individual will proceed to feel pain. Melzack and Wall suggested that, in addition to the spinal cord components, there were additional control mechanisms located in cortical regions of the brain [1]. These regions were thought to be responsible for the effects that emotional factors have on pain perception [1]. Though the gate control theory is founded on a basis of understanding the nature of pain, genetic factors are ignored.

Thirty years after the pain gateway theory, Ronald Melzack’s exposure to amputees suffering from phantom limb pain prompted his inquiry that led to the development of a "Neuromatrix Model” [1]. This model suggests that the central nervous system (CNS) is the origin responsible for creating painful sensations rather than the periphery [1]. The neuromatrix consists of various areas within the CNS which contribute to the signal of pain perception; areas include the spinal cord, brainstem, thalamus, somatosensory cortex, motor cortex, prefrontal cortex, insular cortex, and limbic system, [1]. The neuromatrix hypothesis states that pain is a product of different patterns of signals from these different areas in the CNS; the patterns of signals were referred to as the "neuro signature." Peripheral sensory input, including nonphysical factors, can influence the neuro signature, but cannot create its own signature [1]. This model also acknowledges the notion that pain can be affected by cognitive and emotional factors [1].

Biopsychosocial model 

The most inclusive model of pain is the “Biopsychosocial Model” [1] which provides the most exhaustive explanation of pain etiology. This theory of pain hypothesizes that pain is a result of the intricate interactions between biological, psychological, and sociological factors. It was not until 1977 that the biopsychosocial model was scientifically investigated to explain the etiology of some medical conditions. It supports the notion that the human body cannot be compartmentalized when considering management options for pain. John D. Loeser, an anesthesiologist, was the first person to implement this model [1]. Loeser proposes that four parts needed to be taken into consideration in the treatment of pain: nociception (nociceptive component), pain (sensory-discriminatory component), suffering (motivo-affective component), and pain behaviors (the cognitive behavioral component) [1]. The way we approach pain management today is largely based on Loeser’s four elements of pain, and failure to consider these elements can be considered an inadequate assessment of care. The biopsychosocial model of pain is the closest to incorporating genetic factors.

Despite the progress made in the comprehension of pain perception, there is much that is still unknown. With the current knowledge acquired on gene expression, there has been an increase in attention geared towards pain management and genetics. With expanding knowledge on the subject, more and more studies are being conducted regarding genetic makeup, as well as epigenetic modulation. Many of these genes that have been discovered have not yet been able to be manipulated within the clinical care setting. With many factors internally and externally influencing pain perception, a major goal for pain management is to approach each patient’s pain tailored to that individual. Understanding how these genetic influences are expressed can better aid in this goal. The different pain types, modulators, and registration of the pain are of great importance when considering manipulating the expression of specific genes. Comprehension of the innate nature of the pain process allows for better hypothesizing of ways to manage it.

## Review

Types of pain 

*Nociceptive, Inflammatory, and Neuropathic* 

**Figure 2:**
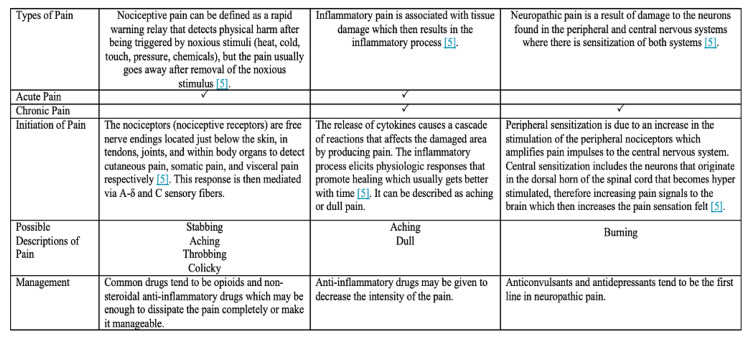
Types of pain – nociceptive, inflammatory, and neuropathic. Reference [5]

As mentioned above, pain is defined as physical suffering or discomfort caused by illness or injury. Figure 2 compares the three different types of pain that can be experienced: nociceptive, inflammatory, and neuropathic [5]. Certain disease processes may involve a combination of the three types of pain making for a challenge in the management, acutely or chronically. Nociceptive and inflammatory pain can be classified as acute pain whereas neuropathic and nociceptive pain can develop into chronicity [5]. Acute pain is defined as pain experienced for a short period of time, usually following surgery, trauma, or other conditions that occur suddenly [6]. Unlike acute pain, chronic pain has no biological function and is defined as pain that lasts beyond the time expected for the disease in question [7]. It is not considered a symptom of a disease process but a disease process within itself, many times occurring idiopathically [7].

Research has shown physiological processes modify pain experience, though in most studies, the anatomy and physiology of pain were investigated by studying cutaneous pain, while most chief complaints of pain in the healthcare setting arise from deep tissues [7,8]. What is known about primary afferent nociceptors comes from studies conducted on cutaneous nerves [7]. These experimental studies on pain neurophysiology provide fair models for acute pain, however, they are poor models for chronic pain syndromes [7]. Little information is provided about the deeper structures, including muscles, joints, and tendons which are most often involved with chronic pain conditions and pain desensitization. Hence, theoretically, the neurophysiology of someone who lives with chronic pain would be significantly different from that of someone who does not, thus hypothesizing that each type of pain would require a different approach. Further studies would need to be conducted to further separate and appropriately distinguish the process of acute versus chronic pain and how genetics play a role in the presentation.

Pain processes

Modulation

The innate process of pain can be divided into four parts: transduction, transmission, modulation, and perception. The four main categories of pain processes have been described in detail in references 8-10 and are beyond the scope of this article. This review will focus mainly on the category of modulation and its further sub-classification. Pain modulation refers to the process by which the body reduces the intensity of the pain as it is transmitted along the pain pathway [9]. At each synaptic cleft mentioned above, the information is integrated and undergoes modulation via inhibitory or excitatory influences [9]. The central modulation of pain perception arises from activating the descending pain-modulating pathways that project via the medulla to neurons in the dorsal horn, particularly the Rexed's lamina II, which controls the ascending information in the nociceptive system [8,9]. The brainstem regions that produce this effect are nuclei within the periaqueductal gray matter (PAG) and the rostral ventrolateral medulla oblongata (RVM) [8,9]. Stimulation of these sites inhibits the activity of nociceptive neurons in the dorsal horn of the spinal cord that initially would produce analgesia [8,9]. Conditioned pain modulation (CPM) is a unique form of the endogenous descending inhibitory pathway [9]. Inhibitory cells, which modulate nociceptive inputs, are recruited by stimulation of non-nociceptive afferents (Aβ-fibers) and inhibited by stimulation of nociceptive afferents (Aδ- and C-fibers) [9]. The function of endogenous pain modulation mechanisms depends on the pathophysiological mechanisms of the type of clinical pain elicited [8,9]. This explains, in part, why individual responses to the same painful stimulus may sometimes differ. Modulation can also explain why the activation of pain neurons and the sensory experience of pain do not always coincide [8,9]. Chemical modulators of pain include endogenous and exogenous opioids, autonomic neurotransmitters (dopamine, norepinephrine, serotonin), and inhibitory amino acids (cholecystokinin {CCK}, gamma-aminobutyric acid {GABA}, and galanin) [10]. The PAG and RVM are sensitive to endogenous opioids as well as to exogenous opioids [10]. There are several categories of endogenous opioids that act on the PAG and RVM: β-endorphins, Met-enkephalins, and dynorphin A [10,11].

Endogenous Opioid Modulation

In the peripheral nervous system (PNS), β-endorphins exert their analgesic effect by binding to opioid receptors, specifically the μ receptor [11]. They are located throughout the periphery and are identified in the central terminals of primary afferent neurons, peripheral sensory nerve fibers, and dorsal root ganglia [12]. Once bound, there is an inhibition of the release of tachykinins. These tachykinins are neuropeptides found in nerve endings throughout the skin, mucosa, and viscera where they behave as potent mediators of vasodilation, plasma extravasation, inflammatory cell recruitment, and pain. Substance P is a tachykinin and is an essential protein involved in the transmission of pain. By inhibiting its release, there is no ascending signal transmitted to the primary somatosensory cortex, therefore no pain is elicited [10,11]. In the CNS, β-endorphins have a similar mechanism of action via binding μ-opioid receptors located in the descending pain circuits, including the amygdala, the mesencephalic reticular formation, the PAG, and RVM [12]. Here they exert their analgesic effect via inhibiting the release of GABA, an inhibitory neurotransmitter, resulting in increased dopamine production, a neurotransmitter associated with pleasure [10-12]. Loss of GABA inhibition of nociception may indicate the development of inflammatory and neuropathic pain [13]. 

In both the CNS and PNS, β-endorphins bind to pre- and post-synaptic nerve terminals, though they exert their effect only when bound to presynaptic nerve terminals. Activation of mu-opioid receptors located within the midbrain generates a downward inhibition of the PAG and nucleus reticular paragigantocellularis. This inhibitory signal is then propagated to 5-hydroxytryptamine and enkephalin-containing neurons that intermingle with the dorsal horn and ultimately inhibit or reduce the conveyance of nociceptive stimuli [10,11]. 

Location of Endogenous Opioid Receptors by Organ System 

Enkephalins are extensively distributed throughout the central, peripheral, and autonomic nervous systems. Met-enkephalin was detected using immunoassay in the CNS within the globus pallidus, hypothalamus, PAG, amygdala, and spinal cord. *PENK* gene expression was recognized in the posterior pituitary and the spinal cord [11]. Glucocorticoids have been shown to directly upregulate the transcription of pro-enkephalin (PENK) [10]. Researchers have presumed that the intensity and duration of the stress response may be due to the role of enkephalins [10-12]. Met-enkephalin has been shown to consist of immunomodulatory effects on the cluster of differentiation (CD)8+ T cells, inhibitory regulatory T cells, phagocytosis in macrophages, the proliferation of CD4+ T-helper cells, and the natural killer cell response [11].

Patients with heart failure have increased pro-enkephalins that directly correlate to the severity of their condition. In experimental models, both beta-adrenergic stimulation and pressure overload increased the expression of enkephalin [11]. In the cardiovascular system, the effects of opioids acutely decrease the heart rate and blood pressure while increasing the myocardial contraction chronically [11]. Respiratory depression is primarily mediated by hypothalamic μ-opioid receptors, and it is the most common fatal side effect of exogenously administered opioids [11]. The main opioid receptors found in the gastrointestinal tract are mu and delta-opioid receptors (located in the submucosal and myenteric plexus, respectively). Activation of mu-opioid receptors inhibits motility in the colon and increases fluid absorption, subsequently leading to constipation [11]. The overall effect of opioids on bodily systems includes decreasing transit time and slowing down the metabolic rate.

Exogenous Opioids

Exogenous and endogenous opioids act on the same receptor types (most opioids target μ receptors), but endogenous opioids do not contribute to the adverse effects associated with exogenous opiates [14]. When exogenous opioid agonists act centrally, unwanted opioid side effects such as respiratory depression, cognitive disturbances, nausea, tolerance, and addiction become a concern. In comparison to endogenous opioids which are delivered to targeted sites of action by immune cells, generally preventing exogenous-like side effects on the central nervous system [14]. Mitigating exogenous opioid side effects is a major frontier in the study of opioid pain modulation.

Modulation by Dopamine, Norepinephrine, and Serotonin

Dopamine has been shown to be important in pain modulation, however, the specific mechanisms by which dopamine modulates pain are generally unclear. Selective serotonin reuptake inhibitors (SSRIs) and serotonin-norepinephrine reuptake inhibitors (SNRIs), while traditionally used as antidepressants and anxiolytics, have clinical efficacy in alleviating neuropathies [10].

Perception

Together, both the CNS and the PNS are involved in the mechanism and pathways of all variations of pain perception. The perception of pain results from endogenous pain inhibition and the facilitatory mechanisms that trigger pain, although activation of nociceptive fibers is not necessary to elicit pain. Phantom limb pain, for example, does not require nociceptive stimuli. As previously mentioned, the neuromatrix is the neural network output of the brain that is genetically determined and then modified by sensory experience [1,9]. The neuromatrix theory of pain can be useful in understanding phantom or chronic pain that is produced. The pain is not produced via sensory input due to tissue injury, inflammation, or pathology of the receptor and nerve fiber, but rather by the output of the neuromatrix [1,9]. The neuromatrix can be influenced by genetics, strong socio-cultural beliefs, expectations, and conditioning behaviors that can determine how pain is modulated as well as explain analgesia placebo. It is noted that factors such as analgesic drugs, distraction, and emotional context also engage nodes of the pain matrix to change the pain experience 

Epigenetic mechanisms of pain

Genetic associations reveal specific biological mechanisms that contribute to a delivered pain response [15]. The presence, or lack of, certain gene(s) hold explanations for the difference in pain physiology. Utilizing the study of epigenetics further expands on the potential for discovering specific genetic modifications that may function as targets for therapeutic interventions [16]. The alteration in gene expression may negatively or positively affect the function of a protein or enzyme associated with pain duration and intensity. There is also significant evidence for the ability of epigenetics to modify general pain types such as inflammatory, neuropathic, visceral, and cancer-related, all of which hold strong evidence for future potential therapeutic benefits [15,16]. This is done through alterations in chromatin structure. Changes in DNA methylation and histone modification such as acetylation, methylation, phosphorylation, ADP-ribosylation, and miRNA expression have been robustly altered in various CNS sites under chronic pain states [15-17]. MicroRNAs (miRNAs) are small, single-stranded, non-coding molecules of RNA which regulate gene expression at the post-transcriptional level [3]. Manipulation of these processes influences pain-related behaviors [17]. These alterations represent ideal mechanisms by which the experience of tissue injury and acute pain can be converted gradually and progressively into pathological processes of neuroinflammation, central sensitization, and ultimately chronic pain syndromes. For example, epigenetic changes appear to mediate the transition from acute to chronic pain after nerve injury [17]. Evidence from multiple studies suggests that chronic pain development is correlated with changes in gene expression within the spinal cord and cerebral cortex [17-19]. Activity-dependent changes in gene expression have been understood to be important for long-term alterations in neural activity [17]. Animal studies have been conducted to investigate the cellular and molecular adaptations of neural activity. Inflammatory pain models used inflammatory agents such as: formalin, capsaicin, or complete Freund’s adjuvant (CFA), whereas neuropathic pain models typically involved direct injury to the spinal cord or nerve [17,18]. Assuredly, both models mimic the key components compromised in the development of human chronic pain, rendering them valuable in the study of gene modifications [16,17,19]. Areas of pertinent modification include the primary afferent, dorsal horn, spinothalamic tract neurons, and several brain regions of the pain matrix [16,17]. Modifications at the dorsal horn (DH) could augment primary afferent activation, facilitating hyperalgesia whereas, in descending pathways, modulation of regulatory brain regions such as the anterior cingulate cortex (ACC), prefrontal cortex (PFC), PAG, and RVM, could lead to allodynia or other sensory deficits [15,17]. Interneuron modulation appears to potentiate allodynia and promote hyperalgesia [17]. 

There is limited research and literature pertaining to epigenetics and how it directly implicates modifications in pain syndromes. Though, there is evidence that injury-induced changes in chromatin structure drive the changes in gene expression and neuronal function [15,16]. When injured, astrocytes, the supportive cells of neurons, undergo changes in gene expression mediated by epigenetic mechanisms that potentially contribute to pain syndromes [17]. These modifications may cause several symptoms in patients, including allodynia, hyperalgesia, anxiety, and depression [15-17].

Disruption of pain modulation can present as a pain disorder. The capacity for sensing pain has developed with a balance between the positive and negative factors that comprise our pain response. This balance allows us to appropriately perceive the pain stimulus without experiencing severe hyperintensity or inadequate response. As formerly mentioned, engagement of descending modulation can facilitate and inhibit pain. Conditioned pain modulation (CPM) describes one of the psychophysical paradigms in which central pain inhibition is tested by means of "pain inhibits pain" [9]. The parallel phenomenon for CPM in animal models is called diffuse noxious inhibitory control (DNIC). CPM and DNIC are both examples of the body’s compensatory mechanism to modulate ascending pain transmission [9]. 

Role of DNA Methylation in Pain

The most recognized mechanism of gene expression modulation is methylation. DNA methylation controls transcription through a mechanism that suppresses transcription factor binding at promoter regions. This is done by a complex consisting of a DNA methylation-dependent binding protein called methyl-CpG-binding protein 2 (MeCP2) [3,17]. MeCP2 is a member of the methyl-CpG binding domain (MBD) containing proteins [3,17]. MeCP2 is a transcriptional repressor that serves in the modulation of activity-dependent gene expression by inhibiting the transcription of specific genes by binding to methyl-CpG sites on DNA [3,17]. DNA methyltransferase (DNMT) is a group of enzymes responsible for the methylation of CpG (cytosine-guanine) dinucleotides and inhibits gene transcription by blocking the accessibility to a transcriptional activator [20]. Phosphorylation of MeCP2 results in its removal from CpG promoter regions that have been methylated, which leads to enhancement of gene expression [17].

Rett syndrome is a developmental disorder seen almost exclusively in females; affected males usually die in utero or shortly after birth. A de novo mutation of the X-chromosome’s *MeCP2* gene seems to cause symptoms to appear between the ages of 1 to 2 years, involving mental retardation with regression in motor, verbal, and cognitive abilities, ataxia, seizures, growth deceleration; and stereotyped handwringing. Rett syndrome’s loss of *MeCP2* function is associated with impaired development of the locus coeruleus, the brain’s main source of norepinephrine [21]. This loss of the brain’s main noradrenergic signaling pathways seems to alter somatic and visceral sensitivity, thereby suggesting a role of *MeCP2* in pain perception [22]. An abnormality in the expression of substance P, a neurotransmitter and neuromodulator of pain, is also proposed [23]. These findings and others suggest that *MeCP2* appears to contribute to the complexity of gene regulation and modulation of transcriptional repression that accompanies pain states, such as the role of the descending serotonergic pathways regulating gene expression through *MeCP2* in the dorsal horn of the spinal cord [24,25]. The clinical significance of these findings is that a low threshold for injuries and chronic pain due to abnormal pain sensitivity should be expected in persons affected with Rett syndrome [22]. 

Findings in rodents have suggested a role of varying degrees of DNA methylation in the regulation of μ-opioid receptor expression within the brain [20,26]. Utilizing a mouse model, Shao and colleagues investigated the methylation of the *DNMT3a* gene and related changes in the μ-opioid receptor (MOR) promoter [20]. The induced nerve injury led to time-dependent upregulation of *DNMT3a* expression which increased methylation of the MOR gene promoter hence decreasing MOR protein expression within the spinal cord [20]. This significant reduction in MOR expression might explain why opiates have limited efficacy in neuropathic pain states. However, when administered a DNMT inhibitor (RG108) there was a significant decrease in methylation at the MOR promoter. This led to upregulated MOR expression which then attenuated thermal hyperalgesia in the mice. This exhibits that methylation can be reversible and can possibly aid in generating drugs to treat neuropathic pain [20]. 

Evidence is emerging that opioids may trigger DNA hypermethylation under chronic exposure [26]. Chronic opioid exposure of μ-opioid receptor gene (*OPRM1*) methylation was studied in postmortem acquired brain tissue of 27 former opioid addicts of Caucasian ethnicity [26]. Using DNA methylation and μ-opioid receptor expression analysis, it has been observed that high versus low μ-opioid receptor expression was not associated with the *OPRM1 *methylation status [26]. However, in vitro experiments in human cell lines indicated that the expression of μ-opioid receptors is associated with differences in *OPRM1* DNA methylation, but this DNA methylation differed by a greater magnitude than the difference observed between the high and low *OPRM1* expression in the brain [26]. These results suggest that the pharmaco-epigenetic effects of chronic opioid exposure are theoretically possible but are unlikely to exert a major role in brain-specific μ-opioid receptor expression.

Regional pain syndrome, also known as complex regional pain syndrome (CRPS), is defined as a painful limb-confined condition occurring specifically after traumas [27-29]. Wang et al. focused on the first relay station, the dorsal root ganglion (DRG), as a critical contributor to the pathogenesis of this syndrome and discussed its potential in being a target for probable analgesia treatment [27,28]. Complex epigenetic modifications of opioidergic gene expression in the DRG due to nerve injury may underlie the genesis of CRPS [27,28]. Bruehl et al. discuss more specifically how DNA methylation profiles are associated with CRPS through convoluted mechanisms [29]. In sum, the cause of this syndrome is due to a complex interaction among tissue injury, nerve injury, and inflammation alongside the mediation of epigenetic factors making for a difficult treatment regimen [27-29].

Role of Histone Modification in Pain

**Figure 3:**
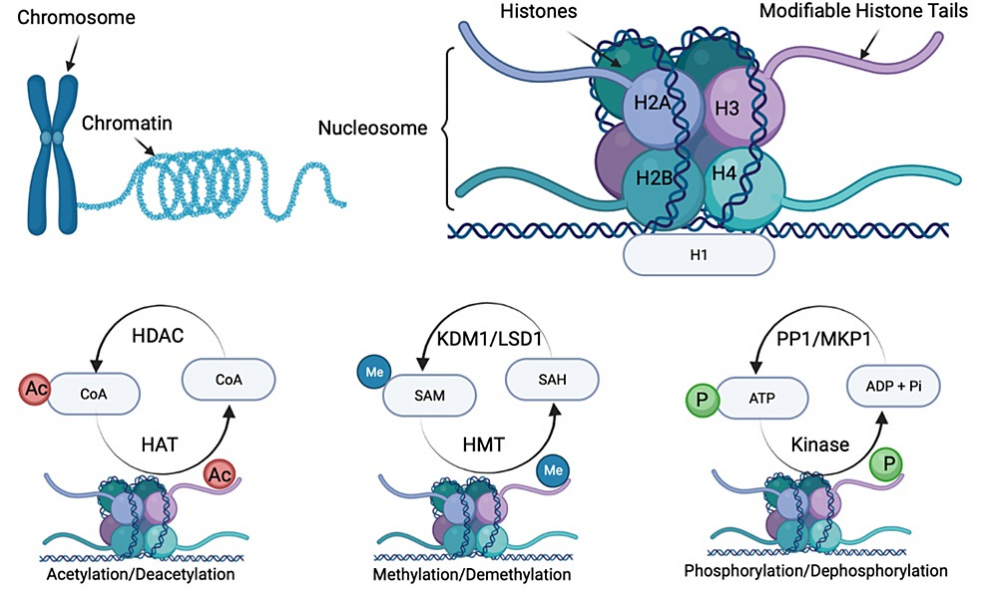
Role of histone modification in pain. Histone modification is the main process by which environment-gene interactions (epigenetics) alter the expression of genes and hence the synthesis of the proteins (e.g., enzymes) involved in gene up- and downregulation and pain perception. The critical epigenetic mechanisms in upregulating or downregulating genes are shown as cycles of acetylation-deacetylation, methylation-demethylation, and phosphorylation-dephosphorylation. H1, H2A, H2B, H3, and H4 are the main subunits comprising histone proteins. HDAC=histone deacetylase; HAT=histone acetylase; Ac=acetyl; CoA=coenzyme A; KDM1=lysine-specific histone demethylase 1A; LSD=lysine-specific histone demethylase 1; HMT=histone methyl transferase; SAH=S-adenosylhomocysteine; SAM=S-adenosylmethionine; Me=methyl; PP1=tyrosine kinase inhibitor; MKP1=mitogen-activated protein kinase phosphatase 1; Pi=inorganic phosphate; P=phosphate; ADP=adenosine diphosphate; ATP=adenosine triphosphate.

Aside from direct regulation of gene expression by DNA methylation, histone modification holds great importance in transcription. Nucleosomes are defined as the basic units of chromatin which consist of genomic DNA packaged tightly around eight proteins that make up a histone octamer. Figure 3 depicts the main mechanisms of epigenetic upregulation and downregulation of genes and their protein products by cycles of acetylation-deacetylation, methylation-demethylation, and phosphorylation-dephosphorylation. Histone tails are positively charged protein domains that bind to the negatively charged DNA backbones. This interaction achieves stability of the chromatin and allows for regulation of which genes are allowed to be transcribed. Dense, compact chromatin, called heterochromatin, cannot undergo transcription. Whereas euchromatin, which is not as tightly wound, is part of the genome that is able to be transcribed. Histone tails are highly susceptible to posttranslational regulation forms such as methylation, phosphorylation, ubiquitination, and acetylation which ultimately affect the structure of the chromatin [30,31]. There can be combinations of these post-translational modifications that can give rise to a variety of functional outcomes. This effect of histone modifications is referred to as the histone code hypothesis [30,31]. Histone acetylation is driven by histone acetyltransferases (HATs), which catalyze the addition of acetyl groups to histones to promote gene transcription [3,17]. Histone deacetylases (HDACs) work oppositely to catalyze the removal of acetyl groups from histones, thereby reducing gene transcription [3,17]. HDAC activity may prevent adaptations in gene expression related to nociceptive sensitization [17]. Following nerve ligation, an upregulation of *HDAC1* expression leads to a reduction of histone acetylation within the spinal cord nerve injury. In addition, it induces HDAC-dependent silencing of the *MOR* gene resulting in loss of the pharmacological target for peripheral morphine analgesia. Thus, HDACs upregulate in response to nerve insults, resulting in histone deacetylation and promoting chronic pain development [17,31]. Multiple HDAC inhibitors have been observed to upregulate histone acetylation in the brain and spinal dorsal horn [31]. It is important to note that the administration of HDAC inhibitors has been shown to alleviate nociceptive sensitization of both inflammatory pain and neuropathic pain [31]. HDACs and HATs activity levels are transient and labile posttranslational modifications that rapidly respond to external stimuli [31]. It is clinically important that HDAC inhibitors are currently being studied for the treatment of cancer [17].

There is evidence of neuropathic pain manipulation with acetylation. Upregulation of HATs appears to promote pain perception [31]. In animal models of neuropathic pain, histone acetylation occurring within the spinal cord produces nociceptive sensitization such that various HDAC inhibitors and HAT inhibitors are effective in the treatment of neuropathic pain [17,31]. For example, histone methylation is found to drive gene expression at the DRG during the development of neuropathic pain, thereby suggesting a novel analgesic treatment for neuropathic pain [31]. It is noteworthy that phosphorylation of histones is associated with transcriptional activation of certain immediate early genes (c-fos, c-jun, and c-myc) in response to external stressors, growth factors, and cytokines [31]. This epigenetic modulation affecting a small population of nucleosomes seems to facilitate the fast activation of other chromatin remodelers to dynamically shift the transcriptional balance between gene activation and repression [17,31]. If this mechanism alters the expression of nociception-related genes during initial pain processing, transcriptional changes accompanying tissue injury and inflammation might be prevented [20], thereby suggesting a novel physiological mechanism for modulating the pain response that may also have clinical relevance in a wide variety of pain states.

*Role of miRNA in Pain* 

How certain primary transcripts are processed into mature miRNAs, and the mechanisms by which miRNAs suppress protein translation, are now better understood [3]. miRNAs can bind to protein-coding mRNAs to repress protein expression post-transcriptionally [3]. The potential of miRNAs to regulate pain-related gene expression in the CNS raises the possibility that miRNAs represent a therapeutic avenue in the management of chronic pain conditions [3,32]. For example, evidence has shown disruption of miRNA processing in primary afferent pathways is sufficient to inhibit injury-induced long-term development of chronic pain-related behaviors [17]. There is evidence of miRNA contributing to the etiology of osteoarthritis (OA) [32]. There are abnormally expressed miRNAs that enhance the production of cartilage degrading enzymes, inhibit the expression of cartilage matrix components, increase the production of proinflammatory cytokines, facilitate chondrocyte apoptosis, suppress autophagy in chondrocytes, and are involved in pain-related signaling [32]. Ongoing research on miRNAs has potential implications for the diagnosis and the development of a novel therapeutic strategy for the management of OA. Additionally, the significant difference in miRNA levels in peripheral blood and synovial fluid between OA patients and healthy populations makes them candidates for being used as biomarkers of the disease [32]. 

A role of epigenetics in the biopsychosocial model of pain 

Studies of epigenetic changes in chronic pain mostly examine DNA methylation across racial and ethnic groups. DNA methylation is altered in the glucocorticoid (stress response) receptor gene (*NR3C1*), which has been associated with depression, childhood stress, low socioeconomic status, and chronic pain [33]. DNA methylation of immune cytokine genes has also been associated with chronic stress states; however, little research has been conducted [33]. Thus, DNA methylation changes may play an essential role in the epigenetic modulation of chronic pain in races with a higher incidence of epigenetic alterations. This may explain the more severe and disabling chronic pain in minorities [33].

In a review conducted by Stankiewicz et al., it was noted that the limbic-hypothalamic-pituitary-adrenal axis (LHPA) is the primary circuit that initiates, regulates, and terminates a stress response [34]. Psychogenic stress evokes adaptive changes in the CNS such as changes in behavior, gene activity, or synaptic plasticity within the hippocampus [34]. The most important finding seems to be that stress-induced epigenetic changes can persist long after the stressor has ended and can underlie functional changes in the brain. 

The transient receptor potential ankyrin1 (*TRPA1*) promoter methylation is reported to regulate the pain sensitivity of patients with multisomatoform disorder (MSD) [35]. MSD is characterized by functionally disabling somatic symptoms with chronic pain as the most common complaint and may be precipitated by life stressors or adverse childhood experiences like trauma. Previous studies have shown that receptor methylation of a particular CpG dinucleotide in the promoter region of *TRPA1* is inversely associated with heat and pressure pain thresholds [35]. There was a correlation between CpG-628 and mechanical pain threshold and more specifically, a correlation of CpG-411 with mechanical pain threshold in female volunteers [35]. This experiment demonstrates that higher methylation levels may lead to higher pain thresholds, but the novel finding is that methylation levels were significantly different between patients with severe and non-severe levels of childhood trauma [35]. This suggests that epigenetic mechanisms may contribute to persistent, pathologic effects of stress and supports the notion that there is a linkage between traumatic life experiences and the later development of elevated sensitivity to pain. 

**Figure 4:**
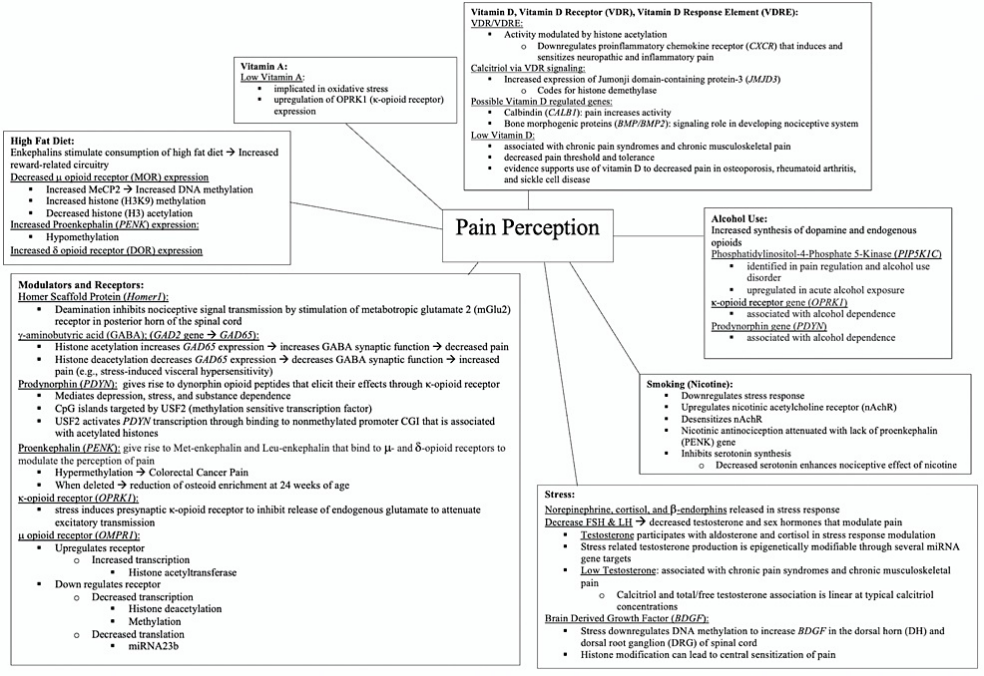
Epigenetic pain perception factors: web diagram of environment-gene interactions and epigenetic modifications which may explain the factors in pain perception. The prodynorphin gene, *PDYN*, codes for the dynorphin peptide which binds to the k-opioid receptor (*OPRK1*) for antinociception and analgesia. The proenkephalin gene, *PENK* codes for Met-enkephalin and Leu-enkephalin binds to m (*OMPR1*) and d (*DOR*) opioid antinociceptive analgesic receptors. High-fat diet decreases the expression of *OMPR1* but increases the expression of *PENK* and *DOR*. Vitamin A deficiency upregulates *OPRK1* for antinociception and analgesia. Vitamin D deficiency decreases pain thresholds and tolerance. The proinflammatory chemokine receptor (CXCR) is downregulated by epigenetic modification of VDR for antinociception and analgesia. Calcitriol modulates vitamin D receptor (VDR) signaling via Jumonji domain-containing protein-3 (JMJD3) for antinociception and analgesia. Calbindin (CALB1) and bone morphogenic proteins (BMP/BMP2) may also be epigenetically regulated by vitamin D for antinociception and analgesia. Alcohol (ethanol) use increases the synthesis of dopamine and endogenous opioids for antinociception and analgesia; dependence may upregulate or downregulate *OPRK1 *and *PDYN* for increased or decreased pain perception. Smoking nicotine upregulates the nicotinic acetylcholine receptors, downregulates the stress response, inhibits serotonin synthesis, and attenuates antinociception and analgesia by depleting proenkephalin. Stress is known to affect pain perception greatly. Norepinephrine, cortisol, and b-endorphins are released in the stress response which initiates the production of testosterone, which may be epigenetically modified through several unidentified miRNA genes. Low testosterone is associated with chronic pain. There is also a linear correlation between calcitriol and testosterone. Upregulation of brain-derived growth factors (BDGF, BDNF) during stress appears to have the potential for acute antinociceptive and chronic pro-nociceptive effects.

Epigenetic mechanisms can be transient, hence making them reversible such as with the histone acetylation discussed previously. This reversibility is popularly sought after when designing pharmaceuticals. There is a paucity of data on ethnicity-related epigenetic factors that can be used to modulate pain, an area in need of further study to advance the development of novel pain treatments. Figure 4 summarizes what is currently known about environment-gene interactions and modifications of receptors, enzymes, ion channels, neurotransmitters, neuromodulators, structural proteins, primary sensory neurons, spinal cord, and other pain-related regions in the brain with the potential for advancing the development of antinociceptive medical therapies.

## Conclusions

A wide spectrum of factors allows for different avenues to be explored in future research on pain perception. Comprehensive and interdisciplinary approaches to adverse life experiences, low socioeconomic status, sex, race, ethnicity, cultural norms, and comorbidities, all of which have been associated with chronic stress and chronic pain, are equally important. Additional opportunities exist to improve pain management through the effects biopsychosocial factors have on epigenetic changes. When assessed in pain-free individuals, these psychosocial variables represent premorbid risk factors for the development of chronic pain but increasing research should focus on resilience factors that may protect against pain.

In addition, there is the social aspect of pain management that is difficult to ignore. Considerable evidence suggests that minority patients are at greater risk for undertreatment of their pain, which could contribute to the greater clinical pain severity observed among members of minority groups. The current limitations in knowledge amongst health care professionals require the medical community to incorporate cultural transformation and competency. This will produce a better understanding of how pain may present as well as how beliefs and expectations about treatment can be linked with practical solutions. While considering all these factors and epigenomic remodeling of the DNA, a personalized pain management plan can be asserted and implemented. Prospective replication and further clinical characterization in greater samples are needed to realize this potential.
